# Application of cell culture technology and genetic engineering for production of future foods and crop improvement to strengthen food security

**DOI:** 10.1080/21655979.2021.2003665

**Published:** 2021-12-02

**Authors:** Rachma Wikandari, Manikharda, Susanne Baldermann, Andriati Ningrum, Mohammad J. Taherzadeh

**Affiliations:** a Department of Food and Agricultural Product Technology, Universitas Gadjah Mada, Yogyakarta, Indonesia; b Faculty of Life Science, Food Nutrition and Health, Food Metabolome, Universitat Bayreuth, Kulmbach, 95326, Germany; c Food4Future (F4F), Leibniz Institute of Vegetable and Ornamental Crops (IGZ), Theodor-Echtermeyer-Weg1, Grossbeeren, Germany; d Swedish Centre for Resource Recovery, University of Borås, Borås, Sweden

**Keywords:** Cultured meat, mycoprotein, genetic engineering, CRISPR-Cas9, Agrobacterium transformation

## Abstract

The growing population and the climate changes put a pressure on food production globally, therefore a fundamental transformation of food production is required. One approach to accelerate food production is application of modern biotechnology such as cell culture, marker assisted selection, and genetic engineering. Cell culture technology reduces the usage of arable land, while marker-assisted selection increases the genetic gain of crop breeding and genetic engineering enable to introduce a desired traits to crop. The cell culture technology has resulted in development of cultured meat, fungal biomass food (mycoprotein), and bioactive compounds from plant cell culture. Except cultured meat which recently begin to penetrate the market, the other products have been in the market for years. The marker-assisted selection and genetic engineering have contributed significantly to increase the resiliency against emerging pests and abiotic stresses. This review addresses diverse techniques of cell culture technology as well as advanced genetic engineering technology CRISPR Cas-9 and its application for crop improvement. The pros and cons of different techniques as well as the challenges and future perspective of application of modern biotechnology for strengthening food security are also discussed.

## 1. Introduction

One of the biggest challenges facing the world today is providing sufficient food for the growing population that is predicted to reach 9 billion by 2050 without harming the environment. In order to meet the future food demands, the overall food production is projected to increase by 70%, in which the production would have to be almost double in the developing countries [1]. The increase production of several key commodities is required. For instance, annual cereal and meat production need to grow by one billion and 200 million tonnes, respectively [1]. On the other hand, the global food supply is threatened by climate change, urbanization, land degradation, water scarcities, and resource-intensive farming system [2]. Today, it has been reported that 720–811 million people are undernourished, and 2.3 billion people do not have access to sufficient food [3]. Therefore, a fundamental transformation of the food system is needed to adequately address this food security challenges.

One part of the solution to meet the goal of ‘zero hunger’ set by the United Nations as one of the sustainable development goals is applying biotechnology. Biotechnology can help to increase food security through a cell culture technology and genomic technology. The cell culture technology enables the growth of foods in the bioreactors thus reducing the usage of arable land. The genomic technology could help crop improvement by marker-assisted selection for precision plant breeding and the genetic modification could introduce valuable traits to crops. Genetic engineering has successfully generated high yield productivity crops, insect, and herbicide-resistant plants which reduce the use of agrochemicals, as well as drought and high salt-resistant crops, which improve agricultural production in marginal areas. In addition, genetic modification could improve nutritional quality of the crops through biofortification. The reduction of the usage of pesticide contributes to decrease the global warming as pesticide is responsible for producing 6.58 CO_2_/kg GHG emissions [4]. In addition, the decrease of land usage would give a positive impact on global warming as large areas of land would need to be reforested around the world to preserve the global temperature rise below 1.5°C. This imply that modern biotechnology could promote sustainable food production.

There are several papers on application of modern biotechnology for crop improvements [5–12]. However, the major paper discusses only genetic engineering of the crops. The cell culture technology is rarely presented. The cultured meat and mycoprotein as the most common example of cell culture technology has also been reviewed by several authors [13–20]. However, there is a scarce information on producing food by plant cell culture technology. This review address advance biotechnological techniques including cell culture technology (animal, plant, and microbial) and genetic engineering as well as the pros and cons of each technique. In addition, the challenge and future perspective of application of biotechnology to improve the food security is discussed.

## 2. Cell culture technology

### 2.1. Overview of cell culture technology

**Figure 1.**
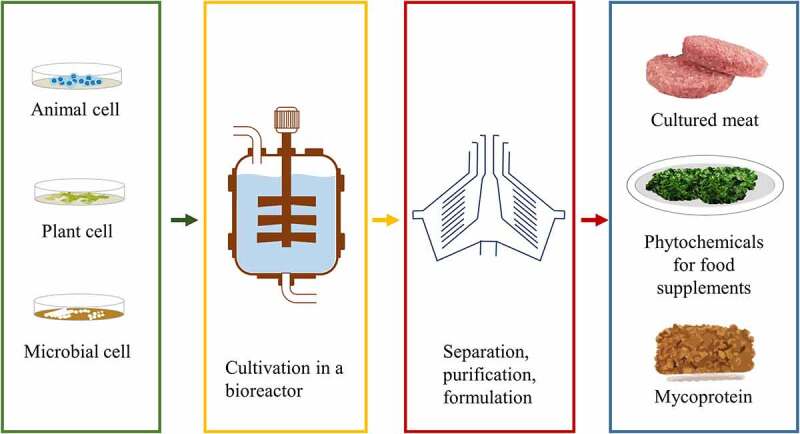
Cell culture technology for food applications

Cell culture technologies form the basis of most alternative methods [21]. They have matured over the last decades. In this review, we will evaluate the animal cell culture (cultured meat), microbial cell culture (mycoprotein) until the plant cell culture, and their prospective application in food technology. Cell culture technology is defined as technology for cell proliferation outside the body of the organisms. Therefore, it offers several advantages including non-seasonal dependent, non-geographical dependent, homogeneity, and controlled production [22]. The cell culture begin with removal of cells from animal or plants and subsequent cultivation in the artificial environment in the reactors (Figure 1). The cell culture requires aseptic and sterile environment to minimize the risk of contamination. Temperature, relative humidity, nutrient, pH, and carbon dioxide level are among factors that should be controlled. The cultivated cells can be grown as adherent cell which are attached in cell culture vessel surface thus forming a layer of as suspended cells which forms a clump for high density cells. Unlike animal and plant cell which are usually grown on synthetic media and use glucose as the main carbon source, microbial cell can be cultivated in various carbon sources including residual biomass. However, these materials require pre-treatment and hydrolysis prior to use. The use of cell culture products for food applications should consider the nutritional compositions and sensory characteristics. For instance, cultured meat should form a structure to mimic the texture of real meat products. Therefore, it requires induction, differentiation of cells as well as nerve-like stimulation and resistance.

Cultured meat has shown a great potential to address food sustainability and nutritional security in near future especially during the pandemic situation [23]. Unlike conventional meat, cultured meat is humane and does not offend the sentiments of animal lovers, hence may satisfy the needs of a larger segment of the society. The technology promises the designer, pathogen-free, ethical, and eco-friendly meat product. But there are many techno-social, economic, and other challenges that have not been resolved yet and play a decisive role in the survivability and viability of *in vitro* technology [23].

Microbial cell culture especially to produce microbial proteins, especially mycoproteins, can substitute partially or entirely animal-based protein foods such as meats [24]. The use of agro-industrial wastes for the production of mycoprotein is multiple targets, especially from environmental aspects. Mycoproteins are important healthy sources of essential amino acids, carbohydrates, vitamins, and also carotenes. Furthermore, mycoproteins can be produced with low total costs, independent of climates (such as flood or drought) and landscape limitations [24,25]. An insight into sensory attributes and consumer acceptance of the mycoprotein products, use and formulation of mycoprotein as meat substitutes had been evaluated and the consumer had a good acceptance for the product. Several reports on the effects of mycoprotein consumption on total blood cholesterol and LDL and HDL cholesterols as well as the impact their effects on satiety, glycemic response, anti-hypertension (ACE Inhibitor), antioxidants have been evaluated where the mycoprotein is a good candidate for the development of functional food [24,26,27].

A novel, sustainably produced, and nutritious food source is needed to feed the growing human population [28]. It is estimated that we will be 10 billion in 2050 and that the current food chain will not be efficient enough to provide nutritious food for everybody. Cellular agriculture, i.e., the utilization of a wide variety of vegetable and fruits plant cell cultures (VFPCs) for the production of agri-cultural commodities could be supplementing current production by farmed animals or crops [28,29]. VFPCs have shown great potential for food purposes, with their relatively high protein, energy, and fiber contents can be established of any plant species and grown in containment independently of environmental factors, e.g., climate or seasonal variations [29]. Due to cultivation in containment, VFPCs are devoid of field-borne pathogens such as viruses and other pests. From the sustainability perspective, the bioreactor-grown plant cell culture biomass can be completely utilized or at least the creation of new waste or side streams is minimized. Thus, VFPCs offers an attractive option in the food production portfolio [29]. Further information about several example of VFPCs is shown in Table 1.

**Table 1.** Some examples of innovative commercially available application for food obtained by plant cell culture [158]

No	Plant Culture	Bioactive Compounds	Purposes	Manufacturer
1	*Euphorbia milii*, suspension culture	Anthocyanins	Food Colorant	Nippon Paint Co. Ltd.
2	*Aralia cordata*, suspension culture	Anthocyanins	Food Colorant	Tonen Co. Ltd.
3	Beta vulgaris, suspension culture	Betacyanins	Food Colorant	Nippon Shinyaku Co. Ltd. N
				Somar Corporation
4	*Echinacea angustifolia*, suspension	Echinacosides	Health Food Ingredient	ABR CBN Plantech IRB Diversa Gesellschaft für Bio- und Verfahrens-technik mbH
5	*Panax ginseng*, adventitious root culture	Ginseng saponin	Health Food Ingredient	CBN Biotech
				Nitto Denko Corporation
6	Wild Ginseng, suspension culture	Ginseng saponin	Health Food Ingredient	Unhwa Corporation
7	*Theobroma cacao*, suspension culture	Cocoa polyphenols	Health Food Ingredient	Diana Plant Sciences
8	*Ajuga reptans*, suspension culture	Teupoloside	Health Food Ingredient	ABR, IRB
9	*Lippia citriodora*, suspension culture	Verbascoside	Health Food Ingredient	ABR

### 2.2. Animal cell culture

#### Overview

The demand of meat was predicted to increase by 72% from 2000 to 2030 [30], that would create a burden on the resources needed [31]. For instance, 7 kg of grain needed to obtain 1 kg of beef and not to mention the time and resources spent on rearing the animals until their age and weight are sufficient enough [32]. The substitution of traditional meat with cultured meat would contribute to the reduction of land use, water resources, and energy use for animal farming [33–35]. It will minimalize deforestation due to creating animal pastures as well as reduction of green house gas emission from animal farming [32,36]. Beside, animal cell culture would also be an answer to growing concern for animal welfare.

Animal cell culture produces culture-based meat, which also known as cell-based meat, cultivated meat, lab-grown meat, clean meat, cultured mea,t or in vitro meat. The product is meat analog derived from culturing and propagating animal cells instead of slaughtering animals for consumption. This breakthrough in food manufacture technology offers a substitute to conventional meat [37]. This development is line with an emerging field refers to cellular agriculture that employs the strategy of manufacturing food from cells.

In 1923, the idea of synthetic food or lab grown food was written by JBS Haldane in his work ‘Daedalus of Science and the Future’ [38] and later in 1930, Frederick Smith mentioned lab grown steaks and chicken breasts as the future prediction of the world in 2030, in which the idea of not wasting time and resources of herding animals to get a piece of steak might be possible [39]. Then, Willem van Eelen, called the Godfather of cultured meat, brought up the concept of lab-grown meat using tissue culture which inspired from stem cell technology in medical school [40]. From his attempt since 1950, he finally received his patent in 1999 [40]. In 1971, Russel Ross published his successful attempt in growing smooth muscle cell grown from the aorta of Guinea pig in vitro [41]. Under a NASA project for Space food in 2002, muscle protein from gold fish (*Carassius* sp.) had been successfully cultivated, harvested and processed as food [42]. Method of producing tissue engineered meat was patented in 2004 by John Vein [43] and the prototype of cell-based meat from bovine skeletal muscle in form of hamburger was presented to the public by Mark Post in 2013 [44,45]. Several studies related to cultured meat are summarized in Table 2.

**Table 2.** Media, cultivation condition and cultured meat products

No	Cell lines	Medium	Scaffold	Bioreactors/ environments	Result	Ref.
1	The ATCC fish fibroblast cell lines of *Carassius* sp	Minimal essential medium (MEM) in Hanks’ salts and minimal essential medium in Earle’s salts both with 10% of FBS, and an adjusted pH of 7.2.	Two dimensional cell cultures in culture dish	The cultures and co-cultured cells were incubated at standard conditions, 23◦ C for 7 days. Observations are made and recorded at 1 day interval	Tissue resembled fish fillets was harvested from cell culture	[42]
2	Isolated satellite cells from cold injured adult chicken fast muscle (pectoralis major) and slow muscle (anterior latissimus dorsi)	Eagle MEM containing with 10% horse serum and 1.5% embryo extract	Two dimensional cell culture in collagen coated culture dish (two dimensional culture techniques)	The cells were incubated at 37°C, fed everyday and maintained for three weeks	Satellite cells from fast and slow muscle both generated embryonic type myotubes in culture, though they differed in peptide arrays.	[159]
3	Myogenic cells from chicken pectoralis muscle isolate	Medium with 85% MEM, 10% horse serum, 5% embryo extract, and penicillin streptomycin, fungizone and gentamicin	Two dimensional culture techniques with coating of 2% gelatin to improve cell attachment	Cultures were preincubated for 3 hr with 25% horse serum in MEM to improve cell and incubated at 37.5°C in a humid atmosphere containing 5% CO_2_.	Muscle tissue is isolated through pipetting techniques, application of tripsin and centrifugation to generate viable myogenic cells for mass and clonal cultures	[160]
4	Bovine myocytes	a substrate consisting of polydimethylsiloxane (PDMS)	culture device consisting of anchors with pillars fabricated using stereolithography	myotubes aligned along its long-axial direction, which contracted in response to electrical stimulation.	millimeter-thick bovine muscle tissues containing highly aligned myotubes which simulates real meat	[161]
5	Newborn piglet semimembranosus muscle satellite cells	For proliferation studies day 1 with MEMα plus 10% fetal bovine serum (FBS) and 10% horse serum (HS), followed by 2 days incubation in serum-free growth medium. In differentiation, 4 days incubation with growth medium including 10% FBS and 10% HS. After 80% confluence, cells were incubated for 24 h in medium with 10% FBS and 1 μM insulin to start differentiation. Then, the cells were cultivated in serum-free differentiation medium (SFDM) for 3 days to turn into myotubes.	Two dimensional culture techniques in gelatin-coated 96-well microplates for 1 day followed by 2 days incubation in serum-free growth medium for proliferation studies. In differentiation studies, myoblasts were seeded in matrigel-coated 24-well plates for 4 days.	The cultured cells were incubated at 37°C with humidified atmosphere of 6% CO_2_ in air. Removing the unattached cells was conducted after 48 hours by refreshing the medium. After 72 h, monolayers were collected.	Satellite cells grown myoblast had been stimulated to perform proliferation and differentiation using the studied conditions.	[162]
6	The isolated satellite cells of pig abdominal thoracic aorta	The Dulbecco-Vogt modification of Eagle’s medium with 3 ml of 7.5% sodium bicarbonate, 1.0 ml of nonessential amino acids and 1.0 ml of sodium pyruvate, and newborn calf serum and 0.5 ml penicillin.	Two dimensional cell culture in falcon tissue culture dish	The cell were cultured at 37°C in an atmosphere of 95% air and 5% CO_2_. The cell confluence achieved after 4 weeks.	Formation of elastic fiber of the muscle was found	[163]
7	The isolated satellite cells of pig skeletal muscle	Dubelcco-Vogt modification of Eagle Medium (DMEM) containing 0.2-M L-glutamine, penicillin, streptomycin, amphotericin, 10% FBS, and 10% donor horse serum (HS).	Two dimensional cell culture in culture dish.	Incubation at 37°C in an atmosphere of 95% air and 6% CO_2_	Validation of cell biopsy technique employing enzymatic digestion, filtration and Percoll gradient centrifugation and cell pooling had been established	[164]
8	Progenitor cells of myoblast and extracellular matrix secreting cells, namely adipocytes and fibroblasts of non human cells	The medium (i.e. Pro-LIF, DMEM/HEPES, or MEM) contains growth factor, cytokines, bioactive agents, nutrients, amino acids, antibiotic compounds, and antiinflammatory compounds. The medium differs in expansion, growth optimization and differentiation stage.	The three dimensional edible scaffold from textured protein with porosity ranging from 20 to 1,000 micrometers.	Incubation at 37°C in an atmosphere of 5% CO_2_	Procedures for three – dimensional cell culturing technique with porous scaffold and the combination of myoblasts, extracellular matrix secreting cell and endothelial cells as well myoblasts differentiation into myotubes.	[165]

The demand of cultured meat was estimated to be growing around 3% per year and even projected to occupy 35% of market share for meat in 2040, while conventional meat was predicted to decrease from more than 90% in 2025 to be around 40% in 2040 [46]. The production cost and the price of cell-based meat also gradually decreased (from $325,000 to $11.36 per patty or $80 per kilogram) as several start-up business from USA, Belgium, Israel, France, Netherlands, and UK, for example Modern Meadows, Mosa Meat, Integriculture, Future Meat Technologies, The Eat Just, New Age Meats, Higher Steaks, Gourmey, Peace of Meat, Meatable and Memphis Meats, attempted to make the food products, like nugget, shawarma, pate, foie gras, burger, meatballs, beef fajita, bacon and sausages, affordable and appealing to consumers compared to animal-based meat [34,44,47–50]. The cultured meat products were expected to be launched in 2021 in form of meatballs, sausages, and burger patties. The market size of the products in total is projected to be around 11.3 million USD [50].

#### Production technology

Animal cell culture technology is developed on the basis of stem cell biology and tissue engineering [44]. The field of stem cell biology concerns with cell that can incessantly propagate unchanging descendants and produce daughter cells with different and more restricted characteristics, while tissue engineering relates to controlling and manipulating the development of tissue into the purposed shapes and functions [51]. The combination of the two fields for example is the application of induced pluripotent stem cells in lab-grown skeletal muscle grafts that initially established in medical field [44].

The raw material of animal cell-based meat are cell lines, suitable medium, essential growth factors for development into appropriate tissue and scaffold to organize the cultured cells within a bioreactor with controlled environment to obtain a large-scale production [32,44]. The technology involves four main components in cell-based meat productions, e.g. obtaining and culturing of cell lines and their co-cultures into muscle and or fat tissue, preparation of culture medium, developing and designing the scaffold or three dimensional structure and construction of the bioreactors [44].

The appropriate cell lines are required with ability to propagate up to certain amount and allow them to differentiate into adult muscle tissue through the modification of culture medium and growth factors. Possible cell lines among others embryonic stem cell (ESC), adult stem cells or progenitor cells, dedifferentiated cells [32] and induced pluripotent stem cells (iPSCs) [52]. One paper had summarized several types of mammalian stem cells [53]. The cells were obtained through biopsy from the animal and proper isolation of stem cells were performed. In the cultured meat production, adult stem cells, including myosatellite cells or myoblast and adipose-tissue-derived adult stem cells, were being exploited [54].

The third components related to the cells organization in a three-dimensional structure, i.e. scaffold material, that supports the function and development of muscle cells, distribution of oxygen, nutrients and signaling molecules, and disposal of cell waste. Unlike two-dimensional cell culture procedures, growing cells in three-dimensional structure has more challenges [55]. The scaffolding materials including natural polymers, animal-derived, and plant-derived, namely, gelatin, collagen, polysaccharides, plant protein, hyaluronic acid, decellularized plant material, fibrin and edible synthetic materials, as well as three-dimensional cell organizing and scaffold fabrication techniques, namely, freeze-drying, gas foaming, selective laser sintering, thermal-induced phase separation, fused deposition modeling, 3D bioprinting, solvent-based extrusion free-forming, solvent casting, electrospinning and stereolithography had been reviewed [52].

The last components is required to condition the cell environment [37] in the bioreactor that can facilitate the cell multiplication and differentiation until they are ready to be processed into meat products [40]. In using myosatellite cells, the induction treatment was performed, first to induce the cells to grow and proliferate up to certain numbers and then induce the cells to merge into multinuclear myotubes and further differentiate into muscle fibers. During the induction and differentiation period, controlled environments, growing substrate or framework, nutrients, and growth factor are critical. While in the differentiation period, the addition of nerve-like stimulation and resistance is necessary [53]. Further processing procedures comprise cell harvesting, medium recovery, waste material removal, and product formulation. The recycle process might involve some steps, namely, membrane filtration, solvent extraction, precipitation, and dialysis. Processing steps in the product formulation depend on the desired end product which include grounding, moisture removal, texturizing, and flavoring [56].

#### Nutrition and safety

Cultured meat is expected to have comparable nutritional content to meat originally derived from slaughtered animals as well as the possibility to modify the content according to the preferred composition or specialized diet for example using co-cultures, genetic modification and supplementation methods [13,23,40,45]. For instance, the fatty acid composition of the meat is expected to be able to contain healthier ones like increasing ω-3 fatty acids and lowering the saturated fats. The promise of culturing technology is the possibility to enrich the products with vitamins, antioxidants, and oxidative stable compounds [57].

The nutritional content of the cultured-based meat depends on the type of cells, scaffold materials used, and cell culture media among others. Muscle cells will have a role as the main source of protein, thus it is projected to be able to provide essential amino acids. On the other hand, fatty acid composition would be affected by mature fat cells or adipocytes. The modification in culture media will largely affect the viability, performance, and nutritional content of the cultured meat. For example, through manipulating the supplementation medium, we would be able to design low fat meat products. The scaffold materials supporting three-dimensional structure for the cell-based meat [48] is also largely affected the nutrition composition in the cell-based meat since the volume of the materials is usually larger in proportion compared to the cultured cells [45].

Other substances that needs due consideration in animal meat are functional amino acids, for instance, taurine, hydroxyproline, carnosine, anserine, and creatine [45,58]. Taurine is reported to have beneficial effect for cardiovascular health and act as cytoprotectant [59,60] and hydroxyproline has beneficial effect on gut health [58]. Anserine and carnosine are potent antioxidant, while creatine is reported to possess positive impact on lean mass, muscle, and cognitive function in older adults [58,61].

The modification of fatty acid profile, especially the increase of essential fatty acid like linoleic and linolenic acid in a co-culture with adipocyte cell technique, is still a challenge since the accumulation of the essential fatty acids in ruminant is derived exclusively from diet [62]. The effect of essential fatty acids supplementation in the culture medium on the growth and lipogenesis of the cultured cells should be further researched [45]. Other possible techniques is combining with genetic modification in the cellular level, as the content of linolenic acid could be increased in transgenic swine that inserted by the fatty acid desaturation 2 gene for a 12 fatty acid desaturase from spinach [44,63]. Otherwise the supplementation of essential fatty acids in later stage of the cell-based meat product would also be possible [45].

Certain compounds from slaughtered animals might not yet present in the current cultured cells technology since many substances that piled up in the muscle are not synthesized by the muscle cells but they are coming from the animal feed that have been processed by other organs or sources. For example, vitamin B_12_, that we seldom find in sufficient amount in plants but available in cattle meat, is exclusively synthesized by microorganism. Since cattle meat is one of rich sources of vitamin B and D, the design of cultured meat is expected to facilitate them as [44,45,64,65]. Minerals like iron, zinc, and selenium are also available in cattle meat, especially the heme form of iron, like in myoglobin, that is easier to be absorbed by the human body and relatively unaltered by natural chelating agent in food [66].

In terms of safety concern, the possibilities of getting food borne pathogen and infectious diseases from cell-based meat are also lower than traditional meat. Other advantages include better protection from the exposure of harmful substances found in poultry and cattle animals like antibiotics, hormones, or heavy metals [40]. Theoretically, culture-based meat is considered safer than conventional meat. Therefore, it would also benefit public health in general.

Sensory properties of food related to texture, aroma, taste, and visual aspects of the products [67] that are defined by the molecular characteristics of the product, such as the type and characteristics of muscles, the fatty acid profile, presence of myoglobin, aroma and taste compounds compositions [36,45]. To be able to replace the position of traditional meat, cell-based meat is expected to have comparable taste or even superior than traditional meat [13]. There are several factors that might make meat from animal cell culture differs from the traditional meat. First, the meat harvested from the the cell culture does not undergo the same biochemical processes that occur during postmortem transformation, therefore it would affect the texture (water-holding capacity and tenderness) and flavor development [45]. The complexity of cell types in traditional meats that contains not only muscle cells and adipocytes but also other cells like vascular network, nerves and connective tissue, would make a difference in the sensory properties [36,44]. The muscle types of cell culture in the current technology also has not yet developed into the adult muscle fibers that is typical of the traditional meat texture [65], whereas color and texture, including hardness, juiciness, and fibrousness are influenced by muscle types [68].

The unnaturalness concern of animal cell-based meat negatively affecting the acceptance of the products [69], even when the target consumers aware of the environmental impacts and animal welfare. Other concerns affecting consumer acceptance are safety, neophobia, healthiness, sensory experience, and price. There are still anxiety about the safety and regulation issues of cultured meats [20,37,44,70–72]. Several attempts on increasing the culture meat’s appeal on the consumers are communicating the advantages of consumption on personal and societal level, emphasizing the superior quality of the final products, and the using the right nomenclature for cell-based meat [44].

#### Challenges and perspectives

Further development of culture meat is expected to be able to imitate traditional meat cuts or steak since only few cell layers can be obtained in the current technology [36,45,65]. The uptake and metabolism of vitamins and minerals by the cultured cells from the media should be further investigated [44]. The nutritional information and health claims for cell-based meat has not yet established for public database and mainly the information available comes from suppositions and the possibility of the current production of in vitro cell cultures though nutrient content of cell cultures can be measured through laboratory assay [44]. Related to environmental and safety concern, there are several things to be considered in the current technology of cell-based meat, e.g. the use of fetal bovine serum, antibiotic or antimicrobial agent, hormone and growth promoters in the culture medium, aseptic packaging and the use genetic engineering [36,44,72].

### 2.3. Microbial cell culture: mycoprotein

#### Overview

Protein is an essential nutrient for our body. It is needed for the growth, repair and maintenance of the good health. It becomes the building block of bone, muscle, cartilage and skin. Meat is one of major protein sources which is highly consumed worldwide. The global demand of meat is projected to 76% increase by 2050 compared to the demand in 2005 [73]. On the other hand, there is a rising concern on meat consumption, which is detrimental to human health, environment, and animal welfare. This situation triggers the development of protein from non-animal cell culture with excellent protein quality and environmental-friendly. Microorganism is an excellent source of protein as it can be produced from a wide range of substrates and required a shorter time for harvesting compared to animal and plants. For instance, yeast and molds biomass can be harvested weekly and for bacteria can be harvested daily, compared to grain crops which are harvested twice a year [74]. Microbial includes protein from yeast, bacteria, algae, and fungi. Among the microbial protein, only fungi particularly filamentous fungi which has a filament to mimic meat fibril. This characteristic is particularly important in developing food as the consumers appreciate not only the nutrition of a food but also the sensory of the product. The single-cell protein produced from yeast and bacteria lacking of this property are mostly developed as feed, food supplements, and food ingredients. Mycoprotein derived from fungi is of interest since it is the first direct and primary protein source for human consumption in the form of meat substitutes. The various mycoprotein products which are available in the market are presented in Table 3. The most well-known example of mycoprotein is Quorn™, which is produced from a filamentous fungus, *Fusarium venenatum* PTA 284. Quorn™ was launched to British market 1985 by Marlow Foods, Ltd, then introduced to US market in 2002 and currently it is available in 16 countries with 70 product variants. Since its first introduction to the market, it attracts public attention as it has more similar texture to meat compared to other meat substitutes. Today, it leads the meat-free sells.

**Table 3.** Table of mycoprotein product in the market

Brand	Company	Products	Sales	Ref.
Quorn	Marlow Foods Ltd	Sausage, nugget, mince, meatball, burger, fillet, etc	17 countries	[15,166]
Promyc	Mycorena	Nugget, ball, burgers	Sweden	[167]
Beyond meat	Beyond Meat, Inc.	Burgers, meatballs, beef, sausage, crumble (pea protein)	the United Kingdom, Germany, Austria, Switzerland and the Netherlands, 80 countries	[168,169]
ABUNDA®	3 F Bio Ltd	Burger	B2B ingredient company	[170]
FermentIQ™	MycoTechnology, Inc.	Meat analogue, burger	United State	[171]
Raised and Rooted	Tyson Ventures	Nugget, burger, sausage (from pea protein isolate)	United State, Europe	[172]
Impossible Foods (investment)	Temasek Holding	Burger, sausage, nugget (plant-based heme is made via fermentation of genetically engineered yeast)	United State, Europe, Asia	[173,174]
Good Catch (investment)	General Mills	Plant-based fish sticks, fish fillets, fish burgers, crab cakes and fish cakes (from peas, chickpeas, lentils, soy, fava beans and navy beans)	The startup will use the funds to expand across North America, Europe, and Asia, it said in a statement, and hopes to launch new fish-free products this spring.	[175,176]

The global market of mycoprotein is projected to be increased in the future. It has predicted that the market of mycoprotein in 2027 will reach US$ 803.9 millions or increase by 45.61% compared to that of the market in 2020. The market will grow at a Compound Annual Growth Rate (CAGR) of 5.5% for the analysis period of 2020–2027. The mycoprotein market in China is estimated to reach US$167.7 millions in 2027 with CAGR of 8.4% over the analysis period of 2020–2027. Besides China, United States, Japan, Canada, and Germany are among the noteworthy geographic markets [75].

The successful commercialization of mycoprotein is linked with technology to culture the fungal biomass in a large scale. The challenge of growing filamentous fungi in a large scale is related to its morphology. The filamentous morphology induces an increase of broth viscosity which rises the shear, difficult for mixing and reduce the rate of oxygen transfer. This challenge can be addressed by introducing air lift bioreactor. Air lift bioreactor uses air injected from the bottom of the reactor for aeration and agitation. Therefore, it offers low shear environment with good mass transfer. Another challenge for production of mycoprotein is forming the meat-like texture, safety, and public acceptance as mycoprotein is a wholly novel food. The filamentous fungi used for making Quorn™ was isolated in 1967; however, it took years for conducting a comprehensive clinical study for safety assessments prior to its commercialization.

#### Production technology

The type of bioreactors for cultivations of microbial cells depends on the morphology and the application of the end products. For instance, bioreactor without mechanical agitation is more appropriate for filamentous fungi cultivation as it does not destroy the structure of the cells and mycelium. In addition, the fungal mycelium hampers the agitation and recovery process. Hence, air lift bioreactor and bubble column which uses compressed air for pneumatic agitation are recommended. The example of fungal cultivation using air lift bioreactor is the manufacturing of mycoprotein Quorn^TM^. The commercial production of Quorn™ begins with inoculum development of *F. venenatum* in a lab-scale bioreactor. The inoculum is cultivated on a medium containing glucose, ammonium and biotin in a 180 m^3^ air lift bioreactor [24,76]. The temperature and pH are set at 28–30°C and 6, respectively [77]. Besides temperature and pH, the dissolved oxygen, nutrient concentration, and growth rate are controlled during the fermentation [78]. After harvesting, the fermentation broth is heated at 72–74°C for 30–45 minutes to reduce the RNA content [77,79]. The next step is centrifugation and vacuum chilling to obtain mycoprotein with 24% total solids. At this point, the mycoprotein in the form of bread dough is ready for further processing into Quorn™ foods. The next processing include mixing, forming, cooking, and freezing. To form a fibrous bundle and creates a meat-like texture, egg albumin is added to the dough to create the bundles. Freezing is also important step to settle the fibrous bundle as the ice crystal growth force the mycelium together. The technology for production filamentous fungal-based foods might be varying in different companies, however there is no information available.

Apart from mycoprotein which can be directly consumed, yeast and bacterial cells are produced as food ingredients or supplements, such as baker yeast and probiotic. In general, production of cell biomass is carried out under aerobic condition, therefore it requires good aeration. For this purpose, different types of bioreactor including air-lift bioreactor, air-sparged reactor, bubble column bioreactor with or without mechanical agitation as well as continuous stirred tank reactor. The production of yeast and bacterial cells is represented by the production of Baker yeast and probiotic powder, respectively.

Baker yeast is one major application of yeast as a biomass for human food. The commercial baker yeast usually uses *Saccharomyces cerevisiae*. The manufacturing process of baker yeast is reviewed by Reed [80]. The yeast can be cultivated in different mediums including carbon source, nitrogen source, minerals, sulfur, vitamin, and trace elements. The carbon sources include cane molasse; beet molasse; maltose from malt-converted grain; glucose, fructose, sucrose, and raffinose from molasse. Meanwhile, the nitrogen source could be ammonia and ammonium salts. The cultivation is commonly carried out in a 150 m^3^ air-sparged reactor with or without agitation and operated in a continuous or fed-batch. The temperature is set at 30°C with the optimum pH at 4.5–5 for up to 20 hours. The fermentation broth typically contains 4–6% of solid. After harvesting, the fermentation broth is then centrifuged to produce a concentrate (yeast cream) with 18–20% solids. The yeast cream can be sold directly to industrial bakery in the form of pumpable refrigerated cream. Alternatively, the yeast cream can be pressed or filtered to obtain 30% solid and the product is called compressed yeast. The compressed yeast is often extruded in the form of blocks and wax wrapped. The yeast cake can also be dried in a continuous belt or in air lift drier to reach 92–96% solids. This product is distributed in a vacuum packed or nitrogen-flush pouch.

The manufacturing of probiotic powder includes inoculum preparation, media sterilization, fermentation, cell harvesting, addition of cryo- and lyo-protectnat, pelletization, and drying [81]. A diverse probiotic strains have been used for commercial production of probiotic such as *L. acidophilus, L. delbrueckii subsp. bulgaricus, L. casei, L. plantarum, L. rhamnosus, L. paracasei, B. lactis, B. animalis, and B. longum* [82]. The growth media contains nitrogen, carbohydrate, salt, and micronutrients. MRS broth is common media for the growth of lactic acid bacteria and *Bifidobacteria*. The frozen seed stock is cultivated in seed bioreactor until reach the desirable quantity followed by transfer to main fermentation vessel or the frozen direct vat inoculation (DVI) containing large number of cells could be directly inoculated on the main fermentation vessel. Different fermentation technology can be applied including membrane bioreactor, continuous or fed-batch fermentation and cell immobilization technology. Meanwhile for the drying, different method and technology which are available include freeze drying, spray drying, fluidized bed, and vacuum-drying [82].

#### Nutrition and safety

Mycoprotein has relatively similar nutrient quality with lower environmental impact compared to animal proteins. With regard to nutrition, mycoprotein has good protein content and digestibility with low fat, and high in fiber, vitamin, as well as polyunsaturated fatty acids. Mycoprotein has high protein up to 45% of its dry matter [24]. The protein digestibility-corrected amino acid score (PDCAAS) very close to the maximum score (0.996) and higher than beef and chicken [83]. The fat content of mycoprotein is four times lower than meat, while the essential polyunsaturated fatty acid content of mycoprotein is eight times higher than meat [24]. Mycoprotein contains 6% of fiber [84] and higher vitamin (39.4 mg/kg) compared to meat (0.61 mg/kg) [24]. Furthermore, mycoprotein is rich in lysine, threonine, and zinc, nutrients that present in low amount in cereal and vegetables [85]. It contains all essential amino acids [24,86]. Several research investigate the functional properties of mycoprotein. It has been reported that mycoprotein is able to lowering serum cholesterol in serum [24,87] and improved glycemic response [24,88]. A recent study reported that there is a number of functional metabolite and proteins from centrate, co-product from the Quorn™ fermentation process. They include a cerato-platanin protein, cell membrane constituents (phospholipids, sterols, glycosphingolipids, sphingomyelins), cell wall constituents (chitin, chitosan, proteins), guanine and guanine-based nucleosides and nucleotides [89].

Mycoprotein received a higher sensory acceptance compared to other meat alternatives such as tofu strips, Tivall stir-fry pieces, Goodbite chicken style, and Vivera vegan stir-fry pieces [90]. The higher overall liking of Quorn is related to its similarity in eating quality to meat particularly the texture. Another study reported approximately 22% participant thought that mycoprotein was meat [91]. Microstructural studies show that the meat-like texture of mycoprotein is resulted from entangled fungal hypha in a fiber bundles, which is cross-linked one to each other by gelled albumen protein [84]. The degree of entanglement is affected by hyphal morphology (branch and length), hyphal aspect ratio, and interaction between hypha and gelled albumin [84].

The safety concern of mycoprotein is related with toxicity and allergenicity. No mycotoxin was detected on the final product of Quorn™ (at LOD of 0.5 ppm) [92], although low level of trichothecenes produced by the fungal strain. This implies that mycotoxin might not be produced during cultivation or destroyed during the processing. There are conflicting results regarding the allergenicity of Quorn™. A comprehensive safety assessments including analytical, animal, human safety data and market information showed that no acute or chronic adverse effects in individuals consuming Quorn™ [93]. Similarly, it suggests that Quorn™ is well tolerated by human with low allergenic potentials [94]. On the other hand, there was 1,752 self-reports on adverse reactions related to allergic and gastrointestinal symptoms. The allergic reactions include urticaria and anaphylaxis, whereas the gastrointestinal symptoms include emesis and diarrhea [95].

#### Environmental concern

In terms of environmental perspective, mycoprotein is more favorable than animal-based protein since it requires lower land and water and produces lower greenhouse gas emission. Water footprint per unit of weight of mycoprotein is 777 L/kg, which is 20 times lower than that of meat (15,415 L/kg) [93]. The land occupation for mycoprotein is 0,00017 ha/kg, meanwhile meat requires 20–29 times higher land occupation (0.0035–0.0049 ha/kg) [93]. The carbon emission released during mycoprotein production is 1.3–2.3 kg CO_2_eq/kg compared to 17–40 kg CO_2_eq/kg for beef [96,97].

#### Challenge and perspective

One challenge for production of mycoprotein in the future is finding alternative substrate and fungi. Currently, mycoprotein is produced from glucose which is derived from food crops cultivated on arable lands. Therefore, alternative sugars derived from non-food crops or non-edible part of food crops such as lignocellulosic biomass could be an attractive approach since lignocellulose is abundant which is not fully utilized yet. Current studies explore a potential to utilize food residue for production of fungi-based meat in lab scale. Gmoser et al. [98] cultivated *Neurospora intermedia* and *Rhizopus oryzae* on a stale bread at 35°C and relative humidity of 95% for 6 days. This cultivation resulted in improvement of protein content, essential amino acids, minerals, vitamins, dietary fibers, and vitamin D2 were obtained. Addition of brewers spent grains on the bread, the main solid by products of beer production, improve the texture of the fungal fermented product to be similar with that of commercial soybean burger. Similarly, Filho et al. [99] developed vegan-mycoprotein concentrate from pea-processing industry by-product. Different fungal strains from Ascomycota had been tested using bench scale air lift bioreactor and *Aspergillus oryzae* showed the most promising fungi with a protein yield of 0.26 g/g. The use of food processing by product to produce protein-rich fungal fermented product is re-introducing by product into food production chain that might contribute to minimize food waste and protein shortage. The biomass yield, techno-economical study, and sustainability of mycoprotein produced by sugars derived from lignocellulose also remain an open area for investigation. In addition, exploration of alternative fungal species or genetic engineering of the mostly used fungi to produce better nutrition and sensory of mycoprotein is an interesting subject for further study.

### 2.4. Plant Cell Culture

#### Overview

Since the foundation of plant biotechnology and the concept of cellular totipotency in 1902 by Haberlandt [29]. VFPCCs, particularly from undomesticated plant species, have been directly exploited for the commercial production of phytochemicals such as pharmaceuticals, pigments, and ingredients for cosmetics and food. In the latter case, the cultivation of plant cells takes place in bioreactors rather than on the field and facilitates fully controlled, aseptic, and year-round production [28].

One of the promising developments of VFPCCs is algae cell culture. The study of different aspects related to the behavior of an algae culture growing in an intensive culture system has gained renewed interest because of the wide fields of application of these photosynthetic microorganisms (Table 4). Algae are viable sources of biological compounds and constitute renewable and environmental-friendly, especially for food. We have already successfully cultured one of the macroalgae cell cultures, *Enteromorpha sp*.

**Table 4.** Major commercial algal products

No	Species	Product	Application
1	*Porphyra*	Nori	Food
2	*Undaria pinnatifida*	Wakame	Food
3	*Laminaria japonica*	Kombu	Food
4	*Spirulina*	Health Food	Nutraceuticals
5	*Chlorella*	Health Food	Nutraceuticals
6	*Laminaria, Macrocystis and Ascophyllum*	Alginates	Thickening, gelling, water retention
7	*Eucheuma cottonii, E. spinosum and Chondrus crispus*	Carrageenans	Gelling, thickening, stabilizing
8	*Gracilaria, Gelidium and Pterocladia*	Agars	Gelling: food and biotechnology
9	*Arthrospira platensis*	Phycobiliproteins	Food colorant, nutraceutical
10	*Dunaliella salina*	β-carotene	Pigment, feed, health supplement
11	*Haematococcus pluvialis*	Astaxanthin	Pigment, feed additive, pharmaceuticals, health supplement
12	*Odontella aurita*	Fatty acids, DHA, EPA, PUFA	Baby food, pharmaceuticals, cosmetics

#### Production technology

Vegetable and fruit plant cell culture (VFPCCs) could also be exploited and evaluated as entirely new food biomass for human consumption. Several VPFPCCs that have been commercialized have been explained in Table 1, such as ginseng plant cell culture to produce ginseng saponin as bioactive compounds, beet cell culture to produce anthocyanins as natural pigments, cacao cell culture to produce cocoa polyphenols as bioactive compounds, and many others example.

Carbon sources explored for plant cell cultivation in the past include monosaccharides and disaccharides as well as sugar alcohols, polysaccharides, and organic acids. There is continued interest in exploring alternative and food-grade carbon sources for heterotrophic plant cell cultivation such as dairy side streams high in lactose, which currently have rather modest recycling or re-use value and thus novel high added-value concepts are constantly looked for in the dairy industry. The media is often supplemented with one or two plant growth regulators. Auxins, cytokinins, and gibberellins are typical growth regulator classes with functions in, e.g., cell division, cell cycle, germination, and flowering [29]. The cultivation of VPFPCCs takes place in bioreactors with the media rather than on the field and facilitates fully controlled, aseptic, and year-round production.

Further explanation regarding the method from the preparation of several cell cultures from berries has been investigated [28]. Based on the previous research for the pretreatment of the cells have been stored on solid medium cryo-preserved in liquid nitrogen. Afterward, the cell suspensions were grown in 250-ml Erlenmeyer flasks containing 60 ml of culture on an orbital shaker at 110 rpm, 24 ± 1°C, and a day-night illumination regime (photoperiod 16:8 h; irradiation 40 μmol m^−2^s^−1^). The Rubus species were cultivated in MS medium containing 3% (w/v) sucrose, 0.1 mg l^−1^kinetin (Sigma, Munich, Germany), and 1 mg l − 1NAA (α-naphthaleneacetic acid;). Lingonberry cells were grown in Woody Plant Medium containing 3% (w/v) sucrose, 2.2 mg l − 1TDZ (Thi-diazuron), and 1.95 mg l − 1NAA(α-naphthaleneacetic acid).

After the biomass is collected from the bioreactors, for further analysis, the cells will be separated from the medium by vacuum filtration. Then, the cells were washed twice with sterile MilliQ water and either used fresh or lyophilized [28]. Further analysis of several bioactive compounds from VPFCCs has been evaluated and potential to be applied as food ingredients and other nutraceuticals applications.

For the study of the cultivation of macroalgae, we already successfully established the method of cultivation and production. The cultivation of the macroalgae of *Enteromorpha sp* has been conducted to investigate further biogenesis of norisoprenoids as the carotenoids breakdown product in the system [100]. The growth of Aonori (*Enteromorpha sp)* is influenced by internal and external factors [101]. The internal factors are related to the life cycle of this green algae. External factors include nutrients, salinity, light, temperature influence the growth of the algae culture. *Enteromorpha sp*. seems to be particularly sensitive to PO_4_–P limitation and NH_4_–N toxicity [101]. Overall, the growth of *Enteromorpha sp*. has been adversely affected by low salinity and it has been strongly suggested that the growth of spore of the algae is strongly salinity-dependent [102]. The light will give influence the cell division of *Enteromorpha compressa* thalli [100]. Temperature is one of the environmental factors which is important for the growth of algae. At 15°C, the thalli of *Enteromorpha compressa* will be maintained in the vegetative period. A temperature of 21°C will induce gametogenesis [100]. Several studies have utilized culture systems to investigate biological processes inside the culture itself [101].

A laboratory cultivation system of algae culture (*Enteromorpha sp*) for further studies of biosynthetic pathways has been established [100]. The comparability of the laboratory culturing system with algae grown in the aquatic environment was proven by comparing previous results obtained from the study of the enzymatic carotenoid cleavage and new aspects, e.g. the analysis of carotenoid-derived compounds in culture media and the headspace was taken into account. Algae culture β-carotene is the major of the carotenoids *in Enteromorpha sp*. On the other hand, β-ionone is the major norisoprenoids that is derived from β-carotene. Further investigation of the enzymatic activity of CCDs was also evaluated. β-carotene as the substrate may be oxidized by the activity of CCDs to produce β-ionone.

#### Nutrition and safety

There are several macro- and micronutrients from VFPCCs that are already investigated from colorants to the bioactive compounds for human health and it also has a claim for its safety to be consumed [28,29]. VPFPCCs can be established and grown in containment independently of environmental factors, e.g., climate or seasonal variations. Due to cultivation in containment, plant cell cultures are devoid of field-borne pathogens such as viruses and other pests. From the sustainability perspective, the bioreactor-grown plant cell culture biomass can be completely utilized, or at least the creation of new waste or side streams is minimized. Thus, plant cell cultures offer an attractive option in the food production portfolio [29].

One example for the promising VFPCCs such as macroalgae culture will be explained. *Enteromorpha sp*. known as Aonori is a green seaweed (*Chlorophyta*) and is well known in Japan because of its dietary properties. From a nutritional point of view, *Enteromorpha sp*. is rich in non-starch polysaccharides, proteins, minerals, and vitamins. Aonori has 16–22.1% of protein, 12.4–18.7% of ash, and 43.4–60.2% of carbohydrate as a percentage of dry matter [103]. Therefore, Aonori is a good source of protein and dietary fiber and is consumed mainly as a seasoning in Japan.

Aonori also contains carotenoids as biologically active substances [104]. The main naturally occurring algal carotenoids are β-carotene, α-carotene, violaxanthin, neoxanthin, and fucoxanthin [100]. The green color of Aonori is resulting from another class of natural pigments, namely, the chlorophylls [103]. Aonori as green macroalgae has a broad aroma characteristic. Several major volatile compounds derived from terpenoids, polyunsaturated fatty acid compounds (PUFA), and sulfuric compounds have been identified [105]. C_13_-norisoprenoids terpenoids such as β-ionone have been found as an important flavor constituent in green alga *Ulothrix fimbriata* and red algae Asakusa Nori (*Poryphyra tenera*) [106].

Besides the function as an impact flavor constituent, it has been demonstrated also that β-ionone plays important role in aquatic ecology, e.g. β-ionone was shown to be a repellent for the freshwater nematode *Bursilla monohystera* and may play an important role as food-finding cues for freshwater herbivores [106,107]. A further study showed that β-ionone has an important role in the carotenoid biosynthesis in fungus *Phycomyces* as well as in *Cyanobacteria* and influences the glucose uptake among *Cyanobacteria* [105].

#### Environmental concern

The nutritional and also dietary intake of plant-based food is generally considered healthier, more sustainable, and safer. However, it will be increasingly difficult to provide such food in sufficient amounts and quality to supply the global population, which will, according to current estimates, require altogether 60% more food in the future by 2050 than produced today. Projections reveal that agricultural land can only be increased by 2% from the current 38% of the total land area. High-intensity agriculture is already a huge environmental burden as it accounts for approximately 20–25% of global emissions and relies on environmentally detrimental fertilizer and pesticides derived from fossil resources [28].

Based on these facts, it becomes apparent that new technologies such as VFPCCs for diverse and also healthy plant-based food production need to be developed to reduce the negative environmental impacts of agriculture including greenhouse gas emissions and soil degradation and to protect the already dwindling water supplies and biodiversity. In addition, a large part of the world population is urban and the share of people living in cities will further increase, which calls for serious consideration of food production in the built environment, too [29].

#### Challenge and perspective

So far, however, the biomass generated by this method has usually been subjected to extraction and the value of the whole material as a foodstuff has not been considered and investigated. To the best of our knowledge, there are only very few scientific studies suggesting the use of VFPCCs or their extracts as food [28,29]. Due to several advantages of VFPCCs, will this technology can be assessed as an eco-friendly alternative method for sustainable production of plant-derived compounds for future food? Further development of method and also mass commercialization will be needed to offer VFPCCs as future food for humans.

## 3. Genetic engineering tools for crop improvements

### 3.1. Overview of genetic engineering tools for crop improvements

**Figure 2.**
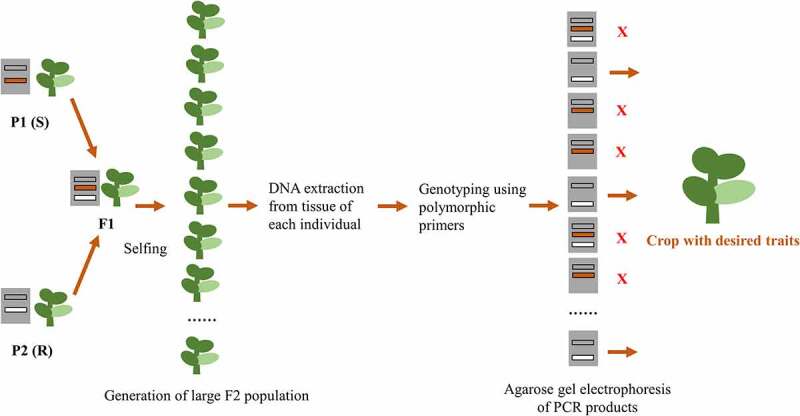
Marker assisted selection for crop improvement

In order to response the rising demand of food supply, crop improvement for enhancement the utilization of wasted lands and increasing crop yield is of importance. The goals of crop improvements are increase photosynthetic efficiency, environmental stress tolerances, insect and herbicide resistance, and responsiveness toward agrochemicals for increasing the crop yield. These goals could be achieved by advanced plant breeding with marker-assisted selection and genetic modification. Marker assisted selection breeding also known as molecular breeding uses DNA marker to determine the genetic makeup of plants, screening of potential parent germplasm, elaboration of genetic linkage maps, identifying gene controlling the desirable trait and selection of quantitative trait [108] (Figure 2). Since the system is genotype-based, it provides higher precision transfer of genomic regions of interest with shorter time requirement, therefore it is considered as a more effective approach compared to conventional breeding which is based on observable phenotypes. Several types of markers are simple sequence repeats (SSR) or microsatellites, single nucleotide polymorphism (SNP), random amplified polymorphic DNA (RAPD), restriction fragment length polymorphism (RFLP), Amplified fragment length polymorphism (AFLP). A number of advance crop biotechnology include application of site-directed nucleases for molecular breeding and insertion or replacement of targeted genes, by passing tissue culture for generating modified plants, gene editing to customized desirable traits, generating multi-generational hybrid vigor, applying rapid domestication for enhancing the variety of the species, as well as insertion of transgenes and synthetic gene clusters [109-].

Transgenic plants that are known as genetically modified plants received foreign genes related to a desired traits or transgenes in their genome through gene transformation process and generate a novel phenotype, which is commonly not available. In general, genetic transformation contains two steps including genetic cargo delivery and regeneration. In genetic cargo delivery, the genes encode the desirable trait that is inserted to vectors which is then injected to the immature plant tissue. Plasmid is among the most common vector. Plasmid often contains marker genes, which will help to select the transformed cell. The markers are usually antibiotic or herbicide resistance genes. The risk possibility of horizontal gene transfer of the antibiotic resistance genes to pathogenic organisms in gut microbiota or herbicide resistance genes to weed become is one of safety concerns in transgenic plant. Moreover, the marker gene is practically of no use once the transgenic crops have been selected. Therefore, to minimize the possibility of marker genes transfer from genetically modified organism or gut microbiota to environment, a marker-free transgenic crops have been developed. Several methods to produce marker-free transgenic plants include co-transformation, particle bombardment or biolistic, site-specific recombinase-mediated marker deletion, transposon-based expelling systems, and intrachromosomal recombination-based excision [110,111]. Another approach is using marker that is not based on herbicide or antibiotic selections. Co-transformation separates transformation of marker and transgene by employing *Agrobacterium*. The particle bombardment uses gold and tungsten to coat the DNA and shut the coated DNA at high velocity to the host cells thus integrating the DNA to the host genome. Since this method do not use vector, thus the transgenes of any size and arrangement can be introduced [112]. However, the disadvantage of both co-transformation using *Agrobacterium* or particle bombardment yields reduction of the resulting plant since these methods integrate the transgenes at random locations in the host genome [113]. In addition, *Agrobacterium* exhibits narrow host and tissue specificity, even within specific cultivars in the sample species [114]. Meanwhile, particle bombardment (biolistic) displays lower yield due to the damages portion of the targeted crops. Therefore, *Agrobacterium*-mediated and biolistic are inefficient, destroy the tissue, or are only effective in a limited number of plant species. On the other hand, nanoparticles are promising alternatives for biomolecules delivery since they are able to pass the plant cell walls without external force and highly tunable physicochemical properties for diverse cargo conjugation and broad host range applicability.

**Figure 3.**
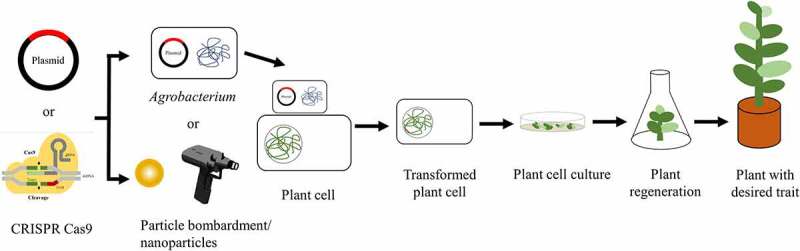
The various techniques and tools for genetic engineering

Genome editing is a powerful technology for editing single to multiple genes in host genome with low cost, high speed, and high efficiency, which provide high, stable, and consistent expression of transgene [115]. Genome editing employs an artificial-engineered nucleases that acts as molecular scissors to cleavage the targeted DNA in a precise and predictable manner [116]. The principal of gene editing is generation of the double-stranded breaks (DSB) at specific site by nucleases and repairment using nonhomologous end-joining (NHEJ) in the absence of donor DNA template or homology-directed repair (HDR) in the presence of donor template [117]. Gene disruption in NHEJ enables insertion or deletion (indels) that results in frame shift mutation and in most cases uses for knocking out the gene in coding region [118]. Meanwhile, HDR enables integration of donor DNA template at the desired locus, which leads to precise gene correction [117]. In order to function site-specific nucleases, the sequence-independent nuclease of restriction enzyme required DNA recognition proteins for binding the desired loci. There are three types of gene-editing tools including zinc-finger nuclease (ZFN), transcription activator-like effector nuclease (TALENs), and clustered regularly interspaced short palindromic repeat and CRISPR-associated protein 9 (CRISPR/Cas 9). ZFN uses a fusion of *Flavobacterium okeanokoitesas* (FokI) as restriction endonucleases and zinc-finger as recognition protein. Each zinc finger protein selectively binds to triplet nucleotide. Similar to ZFN, TALENs also use FokI as restriction nucleases however, for the recognition of protein, it employs transcription activator-like effector which is able to bind specific single nucleotide. Although ZFN and TALENs have successfully applied for editing genome in several plants, however these gene-editing tools face several challenges: (1) difficulties of protein design, synthesis, and validation due to the requirement of protein customization and large-scale screening; (2) time-consuming; and (3) expensive [118,119]. CRISPR/Cas 9 is a breakthrough in genome-editing technology since it offers high specificity and simplicity as well as its ability to do multiplexed gene editing [118]. CRISPR/Cas 9 is derived from natural adaptive immune system mechanism of bacteria or archaea against viral invasion by cleaving the viral DNA with the guide of CRISPR RNAs (crRNAs) (107,108). Therefore, CRISPR/Cas 9 system principally consist of two components, i.e., a single-guided RNA (sgRNA) to identify the targeted loci subjected to cleavage and CAS-9 nuclease, which acts as molecular scissors. Due to its high efficiency, it holds widespread application. The various techniques and tools for genetic engineering is summarized in Figure 3. This section discus application of modern biotechnology for crop improvement for development biofortified crops, insect-resistance crops, as well as salt and drought tolerance crops.

### 3.2. Application of genetic engineering for crop improvement

#### 3.2.1. Biofortification of crops

The problem of malnutrition, especially in developing countries, is characterized by the lack of food consumption in terms of amount and diversity [120]. Malnourished ones usually survive on diet mostly of staple foods like cereal grains that poor in essential micronutrients. To address the problem, food fortification is targeted to alleviate malnutrition and improving overall health and well-being of the population. The concept of food fortification is augmenting or enhancing the content of essential micronutrients or other functional compounds in foods. However, the strategy was rendered inefficient due to poor management, problematic supply-chain networks and the subsistence agriculture that is common in rural areas that makes the agricultural products in those area are hardly processed industrially before reaching to consumers’ hand [121].

An alternative solution that is more appealing to solve the problem is biofortification because the enhancement of nutrients is directly at the level of plant source, without having to modify the processed food products [121,122]. This approach mostly aims rural area where home processing and consumption of staple foods are dominant [123]. Biofortification approach refers to the process producing food crops with enhanced content of micronutrients or other functional compounds, through genetic engineering, conventional breeding [120], or using nutrient-rich fertilizers (agronomic biofortification pathway) [121,124]. There are three steps of micronutrient bioavailability concerning agronomic biofortification pathway: soil to plant, plant to food, and food to humans. Agronomic pathway offers a prompt and effective result to improving micronutrient content in crop, however, genetic biofortification can be considered having less expense in overtime [125].

Fortification of rice and cereal flour with vitamins and iron has been started in 1930 and in the late 1990s the first transgenic rice with daffodil gene has been successfully demonstrated the possibility of provitamin A biosynthesis in non-carotenoid plants using microprojectile bombardment [126] and *Agrobacterium*-mediated transformation, which later improved to increase the provitamin A content [127]. Then in 2004, the cost-effectiveness of biofortification of golden rice in Philippines has been assessed [128], followed by biofortification of staple foods in India [122]. WHO had published fortification guidelines in 2006, and in 2011 the World Bank recommended the implementation of food fortification to G20 countries (Argentina, Australia, Brazil, Canada, China, Germany, France, India, Indonesia, Italy, Japan, Mexico, Russia, Saudi Arabia, South Africa, South Korea, Turkey, the United Kingdom, the United States, and the European Union). In 2016, the metabolomic and regulation study for Golden Indica Rice had been performed and in 2019 the utilization of Golden Rice in food and feed processing had been implemented in Philippines [124]. Studies have evaluated that the problem of nutrient malnutrition in children and women in rural areas, especially those from lower income families, were relatively alleviated through biofortification [129].

The important safety issue to be communicated to consumers is that no high risk of direct adverse impact to human health is suggested investigative studies on biofortification. The process of transgenic technology will not develop allergens, unless the modification involves genes for allergens. The probability of increased gene expression for allergens were also reported to be low. The agricultural biodiversity should also be sustained to avoid the negative effect on the environment and the plants [124]. Precaution should be taken in biofortification via agronomic pathway, especially avoiding over fertilization due to the risk of causing toxic effect or decrease in quality or quantity of the yield [130]. The issue regarding toxicity and excess micronutrients intake also have been tackled through monitoring and control of the frequency and dosage [129].

Some bioengineered agricultural commodities are golden rice (in Philippines, China and India), cassava (Brazil), potato (in North America), tomato, sweet potato, maize, broccoli, mustard oil (in India), and apple (in New Zealand) [122,129,131]. For golden rice products, studies in Philippines evaluated that consumers were relatively ready to buy, especially after they receiving information about the benefit. A meta-analysis study concluded that consumers had developed positive preference toward the products of crop biofortification using genetically engineered method and even willing to pay higher price compared to the common products [122,129].

Problem concerning intellectual property of the biofortified seeds has been reported to complicate the dissemination of the products [129]. Other issue related to commercial products are labeling the GM and biofortified crops [131]. Biofortification as a strategy to address malnutrition could not act as a standalone alternative but supported and influenced by other interventions, for example, considering the fortification of food products in food processing level to avoid excess intake of nutrient and variation of food in one’s diet. The education of biofortification and the dietary choice is still needed in order to accelerate the implementation of the strategy and maintain the sustainability of field productivity, crop quality, and human health in general [122].

#### 3.2.2. Insect resistance crops

Insect pests adversely damage plant growth and development directly and often indirectly by transmitting pathogenic viruses eventually leading to significant losses in crop yields that will give a negative effect on the productivity of the plants. The prevalent agrochemical-based control methods are cost-intensive and environmentally hazardous. It also negatively affects non-target insects such as pollinators, bio-control agents and encounters gradual inefficacy due to the evolution of insecticide resistance in the future. In many instances, the scope of breeding insect-resistant crops is limited primarily due to the non-availability of well-characterized resistance sources within the crossable gene pool. In mitigating this bottleneck several efforts aim for accessing genes from wild relatives and uncharacterized accessions of crop plants. However, much success could not be achieved because of poorly understood genetics of the resistant trait in uncharacterized accessions Alternatively, a transgenic approach has been used for the introduction of insect resistance genes from other distant sources into the crops, for example, Bt genes of bacterial origin [132]. Many excellent accounts of the economic, environmental, and health benefits of insect-resistant transgenic crops have been published [133]. Several research has been investigated especially in grain commodity, if the insects are not controlled, then losses can be 20–80% within a few months after harvest [132].

Genome-editing technology has been successfully applied in a diverse range of organisms including insects for many years. However, recent advancements in the precise application of this technology. Until now most of the research are hovering around optimizing and fine-tuning the components of CRISPR/Cas methods in individual crops. Nevertheless, the leads from basic researches on plant–insect interactions offer vast possibilities of developing insect resistance using CRISPR/Cas9-based genome editing [132]. The future security of food supply will depend on science providing the tools to allow efficient agricultural production to continue that is sustainable in every sense: will the transgenic insect-resistant plants have a track record of success progressively for achieving better food for everyone as the main goals of food security?

#### 3.2.3. Drought and salt tolerance crops

Drought and soil salinity are major stress that limits food crop production as those conditions reduce both growth and yield of food crops [134,135]. Drought tolerance in crops takes form in the plant’s adaptation to the threat of water deficit in terms of changes in physiological functions and a reduced plant cell water potential so that a sustainable balance between water uptake by roots and water release by shoots can be achieved [135]. While salt negatively affects all plant in all development stages through water deficit, ion toxicity, and ion imbalance [136]. Approximately one third of total croplands is reported experiencing from salt stress [137] and is predicted to increase due to the climate change [138]. Soil salinity also hinders the utilization of marginal lands with high salinity for crop production. Therefore, improvement of drought and salt tolerance is a desirable target to reach the current maximum achievable yields and response the raising food demand in the near future.

There are several biotechnological techniques to increase drought and salt tolerance of the crops include mutation breeding, identification of traits using advance molecular technique, marker assisted breeding and transgenic approach. The first three approaches utilize natural diversity, whereas the last approach involves the insertion of novel genes or genetic modification to generate the transgenic plant.

In mutation breeding, a chemical or radiation is applied to alter one or more major traits. The traits modified on developing drought resistance crop are the ones related to plant access to water, minimizing water loss in evapotranspiration and maximizing water use efficiency [135]. Mutation using *N-methyl-N-nitrosourea* in salt-sensitive rice cultivar Taichung 65 resulted in two salt-tolerant mutants M3 with survival rate of 83 and 90% in 0.5% NaCl [139,140]. Identification of traits using advance molecular technique utilizes quantitative trait loci (QTL) mapping. The QTL mapping helps to predict the location of the chromosomal region, which influence the variation of quantitative traits. DNA markers are recently used in the marker-assisted breeding for assessing the inheritance of abiotic stress. Several types of DNA markers include RFLPs, RAPDs, CAPS, PCRindels, AFLPs, microsatellites (SSRs), SNPs, and DNA sequences [139].

Genetic modification approaches in developing drought or salt tolerance crops include regulation of gene expression involved in the plant drought resistance or salinity tolerance mechanism, or insertion of particular transgene to the targeted crops. The mechanism of drought resistance in plants involves osmotic adjustments, osmotic potential, relative water content, water-soluble carbohydrates, and water use efficiency, among others. While the mechanisms of salinity tolerance of the cells include repelling of the Na^+^ from cytosol of photosynthetic cells by plasma membrane H +- ATPase and SOS1, compartmentalization of Na^+^ into vacuole mediated by tonoplast proton pump and NHX antiporters, blocking the influxed Na^+^ by transporter protein HKT, and excretion of Na^+^ from the leaves by glandular trichomes [141]. Therefore, genetic manipulation on the corresponding protein is one approach to improve the the salt tolerance.

For instance, over expression of AtNHX1 has successfully increased the salt tolerance of Arabidopsis [142], tomato [143], rapeseed [144] cotton [145], soybean [146], and peanut [147,148]. At NHX1 encodes the vacuolar membrane-bound sodium/proton (Na^+^/H^+^) antiporter which reduce the Na^+^ sequestration into vacuole and Na^+^ toxicity in cytoplasm [149]. Li et al. [150] had successfully transferred the AtHX1 using a marker-free FLP/FRT method to generate transgenic cereal crops with improved salt tolerance. In addition, recent study reported that OsGrx_C7 gene plays an important role in modulation of salt stress in rice since it intensifies transporters (OsHKT2;1, OsHKT1;5 and OsSOS1) expression [151]. The dehydration-responsive element binding proteins (DREBs), belonging to the AP2 family are the transcription factors which involves in activation of genes responsible for drought and salt tolerance. Nguyen et al. [152] reported introduction of GmDREB6 transgene DT84 cultivar soybean plants, using Agrobacterium-mediated transformation. The result showed an increase of transcriptional level of GmP5CS thus implies that GmDREB6 could improve salt tolerance. The emerging gene-editing technology, CRISPR/Cas9, has successfully identified genes osmotic stress/ABA-activated protein kinase 1 (SAPK1) and SAPK2, which play a role in salt stress [153]. CRISPR/Cas9 technology offers several advantages such as ability to modify multiple genes simultaneously, easily locating the sites of the targeted genes, simplicity to design, and requirement of only short oligo RNA [154,155].

## 4. Challenges and future perspective

Advancements in tissue culture techniques and the culture-media devoid of cultured meat, microbial cell culture, and also plant cell culture supplements may make large-scale cultured production successful provided several properties including the physicochemical and sensory properties such as color, aroma, texture, muscle cell and adipose cell proportional and palatability are comparable to conventional food. The developments of more sustainable cell lines, balanced flora-based nutrient media, quality scaffolding materials, and large-scaled bioreactors for sustainable production are some of the technical hurdles that need to be addressed to make the cultured cells a viable option for the future [23].

Public acceptance, structural incompetency, and economy of the products are the main areas that need due consideration for the successful acceptance of cultured cells such as cultured meat, microbial cell culture (mycoprotein), and also plant cells in the food market. In the current scenario of Covid-19 pandemics, cultured cells may emerge as a basic need of the food industry. However, complete replacement of conventional food with cultured cells food may lead to adverse long-term impact on agriculture-based economies of developing countries [23].

The plant transgenic food future prospective will be quite promising also in the future.

Today, transformation to produce genetically engineered crops is the fastest and most widely adopted technology in agriculture. The rapidly increasing number of sequenced plant genomes and information from functional genomics data to understand gene function, together with novel gene cloning and tissue culture methods, are further accelerating crop improvement and trait development recently [156]. These advances are welcome and needed to make crops more resilient especially to climate change and to secure their yield for feeding the increasing human population.

Despite the success, transgenic plants face to challenge both technical and regulatory challenges. For technical challenges, transformation remains the bottleneck because many plant species and crop genotypes are recalcitrant to established tissue culture and regeneration conditions, or they show poor transformability. Improvements are possible using morphogenetic transcriptional regulators, but their broader applicability remains to be tested in the laboratory. Advances in genome editing techniques such as CRISPR CAS9 and direct, non-tissue culture-based transformation methods offer alternative approaches to enhance varietal development in other recalcitrant crops [132,157]. For the regulatory challenge, regulation of GM crop cultivation and trade is very diverse depending on the country or region. Several countries have long history experience with GM crops thus more flexible the GM crops.

## 5. Conclusions

The future food production will face challenges, *i.e*., providing sufficient food for the growing population, dealing with climate changes, decreasing agriculture land, and fresh water shortage. Substantial advancement in biotechnology through cell culture technology and modern genetic engineering tools are promising approaches to meet the challenges. Cell culture technology provides the opportunity for production of novel foods, which are nutritious and sustainable. Meanwhile, the advanced genetic engineering generates crops with improved traits in terms of nutrition and resistance toward adverse environmental conditions yielding a higher quantity and quality of the crops. Appropriate application of the aforementioned technology would contribute to meet the sustainable development goals, i.e. end hunger and climate change.

## Highlights

- Cell culture technology and genetic engineering support strengthening food security.
- Cultured meat is promising future foods as sustainable protein source.
- Mycoprotein is nutritious food, sensory acceptable and has low environment impact.
- Plant cell technology is a new approach to consider for bioactive compound production.
- CRISPR Cas-9 is cheaper and more efficient gene-editing method for crop improvement.
